# Recent evidence exploring the associations between physical activity and menopausal symptoms in midlife women: perceived risks and possible health benefits

**DOI:** 10.1186/s40695-015-0004-9

**Published:** 2015-08-11

**Authors:** Kelley Pettee Gabriel, Jessica M. Mason, Barbara Sternfeld

**Affiliations:** 1 Division of Epidemiology, Human Genetics and Environmental Sciences, University of Texas Health Science Center at Houston: School of Public Health -- Austin Regional Campus, Austin, TX USA; 2 School of Public Health, Austin Regional Campus, 1616 Guadalupe Street, Suite 6.300, Austin, TX 78701 USA; 3 Michael & Susan Dell Center for Healthy Living; University of Texas Health Science Center in Houston, Houston, TX USA; 4 Division of Research, Kaiser Permanente Northern California, Oakland, CA 94612 USA

**Keywords:** Physical activity, Menopause, Midlife, Women

## Abstract

Although the health benefits of physical activity are well established, the prevalence of midlife women accumulating sufficient physical activity to meet current physical activity guidelines is strikingly low, as shown in United States (U.S.) based surveillance systems that utilize either (or both) participant-reported and device-based (i.e., accelerometers) measures of activity. For midlife women, these low prevalence estimates may be due, in part, to a general lack of time given more pressing work commitments and family obligations. Further, the benefits or “*reward*” of allocating limited time to physical activity may be perceived, by some, as too distant for immediate action or attention. However, shifting the health promotion message from the long term benefits of physical activity to the more short-term, acute benefits may encourage midlife women to engage in more regular physical activity. In this article, we review the latest evidence (i.e., past 5 years) regarding the impact of physical activity on menopausal symptoms. Recent studies provide strong support for the absence of an effect of physical activity on vasomotor symptoms; evidence is still inconclusive regarding the role of physical activity on urogenital symptoms (vaginal dryness, urinary incontinence) and sleep, but consistently suggestive of a positive impact on mood and weight control. To further advance this field, we also propose additional considerations and future research directions.

## Introduction

The aging of the baby boomer cohort, born in the United States (U.S.) between mid-1946 and mid-1964 [1], has resulted in increased interest in strategies to optimize the health and well-being of midlife adults (ages 45 to 64 years). Indeed, research efforts specifically targeting midlife women, in particular, has increased exponentially in recent years. This interest may be due, in part, to the relatively recent recognition that sex differences exist, not only with regards to the incidence and/or prevalence of various health outcomes, but also with the prevalence of health behaviors (e.g., not meeting physical activity guidelines) that increase one’s disease and/or mortality risk.

Physical activity is a viable strategy to reduce the burden of chronic disease and disability. Strategies to increase physical activity at the individual- and population- level are particularly appealing given the strong evidence demonstrating the multiplicity of health benefits, including reduced risk of premature death, coronary heart disease (CHD), stroke, hypertension, hyperlipidemia, type 2 diabetes, metabolic syndrome, breast and colon cancer, and depression [2]. Further, since physical activity is a behavior and, thus, is modifiable, it is an excellent target for health promotion interventions focused on prevention.

Yet, despite the well-established health benefits of regular, habitual physical activity, few midlife women are accumulating sufficient levels to meet physical activity guidelines. Current U.S. based aerobic guidelines encourage: (1) ≥150 min per week of moderate intensity physical activity, (2) ≥75 min per week of vigorous intensity physical activity or (3) an equivalent combination of moderate and vigorous intensity physical activity (MVPA) [3]. The guidelines also recommend that adults participate in muscle-strengthening activities, across all major muscle groups, on ≥2 days per week. Based on 2013 Behavioral Risk Factor Surveillance System (BRFSS) data [4], 28.2 % (Standard Error (SE) ± 0.41) of women aged 45 to 54 years met the aerobic physical activity guidelines only, 6.0 % (SE ± 0.20) met muscle-strengthening guidelines only, and 14.8 % (SE ± 0.30) met both the aerobic and muscle-strengthening guidelines. Among women aged 55 to 64 years, the prevalence estimates for meeting guidelines were similar: 28.5 % (SE ± 0.37), 5.6 % (SE ± 0.19), and 13.7 % (SE ± 0.26) met aerobic guidelines only, muscle-strengthening guidelines only, or met both guidelines, respectively [4]. In the 2003–04 and 2005–06 cycles of the National Health and Nutrition Examination Survey (NHANES) [5], physical activity levels of a U.S. representative sample were also directly measured via accelerometers. The prevalence estimates for meeting physical activity guidelines were strikingly lower than those obtained in BRFSS using self-reported methods (as reported above). Using NHANES 2003–06 accelerometer data, only 26.7 % (SE ± 2.4) and 18.0 % (SE ± 2.6) of midlife women, aged 45–54 and 55 to 56 years, respectively, met aerobic guidelines. To be consistent with the wording of the *2008 Physical Activity Guidelines for Americans* [3], meeting physical activity guidelines was defined as *any* accumulated time (minutes per day) spent above the moderate- intensity threshold (1952 counts per minute [6]), and did not necessarily occur in prolonged activity bouts. It is important to note that these low prevalence estimates may be due, in part, to functional limitations that emerge during midlife. Previous studies have found that 20–40 % of midlife women reported moderate to severe physical limitations [7, 8], which could serve as a significant barrier to engaging in sufficient, higher intensity physical activity to meet current physical activity guidelines.

Previous studies have suggested that another barrier to engaging in sufficient physical activity is a *“lack of time”* [9–11]. This is certainly a tangible barrier for midlife women given that during this stage of adulthood, women often find themselves “*sandwiched*” between caring for both dependent children and aging parents [12]. In addition, many midlife women are working outside the home; according to 2013 BRFSS data [4], 56.6 % (SE ± 0.46) and 43.1 % (SE ± 0.41) women aged 45 to 54 and 55 to 64 years, respectively, reported being employed for wages. Additionally, self-employment was reported in 8.6 % (SE ± 0.28) and 7.1 % (SE ± 0.21) of women aged 45 to 54 years and 55 to 64 years, respectively. Work demands and family obligations, therefore, frequently compete with the desire for leisure time activities such as physical activity, given the limited amount of time (i.e., leisure time) available during a day.

Principles of behavioral economics posit that decisions about being physically active involve trade-offs relative to fixed resources [13]. Thus, allocating time to recreational physical activity, given other competing demands, may be perceived as a “*risk*” for midlife women. However, individuals may undertake this risk, if the perceived “*rewards*” are sufficiently adequate and/or valued. The risk of developing the top three leading causes of death in women (i.e., coronary heart disease, cancer, and stroke) [14] increases with age, with risk escalating after age 65 [15, 16]. While midlife women are at immediate risk for developing these conditions, they may not perceive that a reduction in disease risk, that may be manifested in the future, is an adequate reward given the immediate risk of needing to allocate ≥ 30 min per day for physical activity in their already full schedules.

One potential strategy to alter the risk/benefit ratio and increase the prevalence of midlife women meeting physical activity guidelines may be to target health promotion messages centered on the benefit of physical activity for more acute health outcomes or concerns, such as a reduction in- or relief from- menopausal symptoms. A 2005 review paper by Woods and Mitchell [17] summarized the prevalence of menopausal symptoms from published community-based longitudinal studies of the menopausal transition by Staging Reproductive Aging Workshop (STRAW) criteria, whenever possible. The prevalence of reported vasomotor symptoms ranged from 6 to 13 % in the late reproductive phase to as high as 79 % among postmenopausal women. The prevalence of reported vaginal dryness ranged from 3 % of women in the reproductive stage to 47 % among women who were 3 years postmenopausal. According to data from the Study of Women’s Health Across the Nation (SWAN), Sampselle et al. [18] found that 57 % of study participants reported urinary incontinence with 15 % reporting it as moderate and 10 % as severe. The prevalence of reported sleep disturbances ranged from 31 % of women in the reproductive phase to 45 % among women who are 3 years postmenopausal. With regards to reported depressed mood symptoms, the prevalence estimates ranged from 19 to 29 %. Several other longitudinal investigations have reported significant increases in mean body weight and other markers of adiposity (e.g., waist circumference and fat mass) during the menopausal transition [19–22]. In addition to the moderate to high prevalence of reported menopausal symptoms in mid-life women, other studies [23, 24] suggest that these symptoms may persist for a substantial portion of the menopausal transition. For example, a recent (2015) longitudinal SWAN analysis found that vasomotor symptoms persisted for a median duration of 7.4 years [25], and even longer among some demographic groups such as Black women.

Therefore, if women were convinced that physical activity would improve their most salient and disturbing symptoms, they might accept the “*risk*” of allocating valuable time to be physically active in exchange for the “*reward*” of symptom relief. They would also, as a secondary, longer-term reward, gain additional benefit in relation to chronic disease and disability prevention. The purpose of this paper is to evaluate whether this health promotion message is tenable by reviewing the recent literature (i.e., past 5 years) reporting on the effect of physical activity on menopausal symptoms. The selection of menopausal symptoms included in this review were based on the prevalence estimates reported by Woods et al. [17] We also provide commentary on the strengths and limitations of the existing research, and propose future research directions.

## Review

The recent literature, published with the past 5 years (i.e., January 01, 2010 to February 28, 2015), exploring the association of physical activity with menopausal symptoms that are frequently reported by midlife women was reviewed and summarized [26]. Menopausal symptoms targeted in this literature review include those related specifically to hormonal changes that characterize the menopausal transition (i.e., vasomotor symptoms, including hot flashes and night sweats, and vaginal dryness) and more general symptoms that are characteristic of midlife and/or the normal aging process (i.e., urinary incontinence, sleep quality and/or sleep disturbances, psychological distress, and weight gain). While all selected studies for this review are included in the summary tables, investigations utilizing prospective cohort-, quasi-experimental-, or experimental- study designs are highlighted in the text. Studies were not included in this review if more general symptom categories (e.g., urogenital symptoms versus urinary incontinence) were ascertained and/or reported by study investigators. This literature review summarizes the major findings from 14 cross-sectional studies [27–40], 2 longitudinal studies [41, 42], 7 prospective cohort studies [43–49], 1 non-randomized intervention studies [50], and 9 randomized controlled trials (RCT) [51–59] (see Tables 1, 2, 3, 4, 5 and 6).

**Table 1:** Selected studies of physical activity and vasomotor symptoms (includes hot flashes and night sweats)

Reference	Sample	Physical activity measure	Menopausal symptom measure	Other measures	Detailed findings	Summarized findings: observed association			
						Null	Positive	Negative	Mixed
Cross-sectional studies									
Canário et al. 2012 [27]	Population-based sample of 370 women from Natal, Brazil aged 40-65	International Physical Activity Questionnaire with three categories of classification: sedentary, moderately active and very active (vigorous)	Blatt–Kupperman Menopausal Index with three categories of classification: mild (≤19), moderate (20–35), or severe (>35)	Socio-demographic and behavioral characteristics	Bivariate analysis revealed a statistically significant inverse association between physical activity and hot flashes			x	
Haimov-Kochman et al. 2013 [28]	151 healthy women aged 45–55 who attended the menopause clinic at the Hadassah Hebrew University Medical Center (Jerusalem, Israel)	Physical activity was quantified by self-reported frequency of exercise (1–7 times a week), and categorized into 3 groups: 1–2; 3–4; 5–7 times per week	The Greene climactic scale, estimates include total score. Subscores for psychological, somatic/physical, sexual, and vasomotor symptoms also reported	Demographic, anthropometric, and lifestyle (behavioral) variables	There was no association between physical activity frequency and the vasomotor subscale	x			
Kandish et al. 2010 [29]	Female employees at a Mid-Western University were invited to participate in an on-line survey. The analytic sample included 196 women aged ≥40 years that did not smoke or use hormone therapy	Usual physical activity per week reported via 30 min intervals of aerobic and strength activity. Intensity of activity was reported as mild, moderate, or heavy	Usual daily frequency and severity (10-point scale, ranging from ‘very mild’ to ‘very severe’) of hot flashes were ascertained	Socio-demographic characteristics, alcohol and caffeine consumption	Adjusted analyses, suggested higher frequency of aerobic physical activity significantly increased the frequency of hot flashes. Yet, higher intensity of aerobic physical activity was associated with decreased frequency and severity of hot flashes				x
Mansikkamäki et al. 2015 [30]^a^	Random sample of 5000 women born in 1963 was obtained from the Finnish Population Register Centre. Analytic sample included 2606 women aged 49 years old that responded to a postal survey in 2012	A single item pertaining to usual exercise (frequency and duration) per week during past 12-months. Women were classified as ‘active’ if they reported ≥ 150 min per week of moderate intensity or ≥75 min of vigorous intensity, with strength training and balance training	Women’s Health Questionnaire addressing nine domains of physical and emotional experiences, including vasomotor symptoms	Socio-demographic factors, anthropometrics, self-rated health	In the unadjusted models, inactive women had a higher odds of vasomotor symptoms (POR 1.19; 95 % CI: 1.03–1.36). However, after adjustment for BMI and education level, results were no longer statistically significant	x			
Moilanen et al. 2010 [31]	Participants drawn from Finnish Health 2000 Study (*n* = 7,977), data collection included a home interview, 3 self-administered questionnaires, and a clinical exam. Analytic sample included 1427 women, ages 45–64; known menopausal status) who completed the home interview, first questionnaire	Physical activity was assessed via a single item on the questionnaire, “How much do you exercise or strain yourself physically in your leisure time” with four response options ranging from ‘sedentary’ (reading, watching television) to ‘competitive sports’. Participants were classified based on low, moderate, and high physical activity	Severity of general symptoms, including vasomotor symptoms, were assessed via two items on the questionnaire	Socio-demographics, health behaviors, anthropometrics, menopausal status and hormone therapy use	Low active women reported significantly more vasomotor symptoms (β = 0.18; 95 % CI: 0.10, 0.27) than the high active group after adjustment for baseline age, menopausal status, education, chronic disease, and hormone therapy use			x	
Pimenta et al. 2011 [32]	Community-based sample of 243 women (Lisbon, Portugal) that reported vasomotor symptoms in the past month; aged 42–60 years old	Physical exercise was assessed using reported frequency and duration of exercise sessions per week. Summary scores were computed using the mean frequency and duration values	Menopause Symptoms’ Severity Inventory was used to assess the frequency and intensity of night sweats through classification on a 5-point Likert scale which ranged from ‘never’ to ‘daily’ and from ‘not intense’ to ‘extreme intensity’. Severity for each symptom was computed as the mean frequency and intensity values	Socio-demographic characteristics, health and menopausal related variables and lifestyle factors	Physical exercise was not associated with perceived severity of hot flashes or night sweats	x			
Tan et al. 2014 [33]	305 Turkish (District of Izmir) menopausal women who went to their primary care physician between August and October 2009	International Physical Activity Questionnaire (IPAQ)-short version. Women were classified as: low, moderate, or high active	Turkish version of the Menopause Rating Scale (MRS), which includes 11 items assessing assess somato-vegetative, psychological and urogenital symptoms; scores range from ‘not present’ to ‘very severe’	Socio-demographic factors, health behaviors, anthropometrics	There was no difference in the reported frequency of hot flashes/night sweating by physical activity groups	x			
Short-term (≤30 days) Longitudinal Studies									
Elavsky et al. 2012 [41]	Community-dwelling midlife women (*N* = 121; age range, 40–60 years) not using hormone therapy for at least 6 months. Prospective monitoring across a 15-day period. The analytic sample included 92 participants that reported a menopausal-related vasomotor symptom (i.e., night sweats or hot flashes) within the last 2 weeks	To examine the acute effects of PA, participants attended a second visit during week 1, where they completed a 30-min moderate intensity exercise bout. Daily PA was also assessed objectively using an ActiGraph (GT1M) accelerometer placed over the participants’ nondominant hip for 15 consecutive days	Hot flash and night sweat data were collected using Purdue Momentary Assessment platform in which participants self-reported hot flashes and night-sweats in real-time using a personal digital assistant (PDA). Objective data were obtained via skin conductance monitoring (Biolog Hot Flash Monitor), a battery-powered, portable device. Participants wore the monitor for 24 h, twice during data collection. In addition to continuous monitoring, participants were asked to flag perceived events	Basic demographic and health history information. Psychological symptoms through questionnaires	An acute bout of moderate-intensity of aerobic exercise decreases both reported and objective and subjective hot flashes				x
					There was no significant change in night sweats as a result of the acute exercise bout				
					Daily physical activity was not associated with reported hot flash frequency. Yet, less fit women reported more hot flashes on days when they engaged in more moderate-intensity physical activity than usual				
					The associations between daily PA and night sweats were not reported				
Elavsky et al. 2012 [42]	24 symptomatic peri- and post-menopausal women not on HT were picked from volunteers who responded to advertisements	Participants used accelerometers across a menstrual cycle or for 30 days if postmenopausal. Accelerometer count data were classified as % time sedentary, and in light, moderate and vigorous 2intensity physical activity (Matthews cutpoints)	Daily HFs were reported using an electronic PDA across one menstrual cycle or 30 days	Socio-demographic and health history. Psychosocial questionnaires, including depression, chronic stress, and anxiety. Reproductive hormones via blood draw	The association between physical activity and hot flashes was statistically significant in half the participants (*n* = 10 of 20). Same day, as well as cross-lagged (effects of previous day’s physical activity on hot flashes the next day), were examined. Yet, the direction and magnitude of the association varied across participants				x
Prospective cohort studies									
Gibson et al. 2014 [43]	Analytic sample included Study of Women’s Health across the Nation (SWAN) participants (*n* = 51); Pittsburgh site, only. At enrollment (1996–97), participants were aged 42–52 years old. Hot flashes were assessed in 2008–09	PA was measured using accelerometer-derived activity counts from the Biolog monitor. The mean activity count in the 10 min before a hot flash were classified as “pre-flash” physical activity. The other data were classified as “control” physical activity. Habitual physical activity assessed via the Kaiser Physical Activity Survey (KPAS)	Self-reported hot flashes were assessed using a portable electronic diary. Physiologically detected hot flashes were measured using Biolog sternal skin conductance monitors	Socio-demographic and health behavior information, anthropometrics, depression & anxiety	There was no relationship of daily physical activity with physiologic hot flashes, self-reported hot flashes, or physiologically monitored hot flashes (not confirmed by self-report). Yet, higher habitual PA, higher BMI, more depressive symptoms and anxiety were associated with higher levels of self-reported hot flashes not corroborated by a physiologic hot flash				x
Gjelsvik et al. 2011 [44]	Analytic sample included 2229 women aged 40–44 years, randomly selected from national survey in Hordaland County, Norway. Baseline data were collected in 1997–98 and follow-up occurred every second year and continued to 2010	A short follow-up questionnaire included items pertaining to physical exercise. Participants were classified as inactive based on <1 h hard activity and/or <2	A short follow-up questionnaire included items pertaining to the reported frequency (‘daily’ to ‘never/almost never’) and burden (‘very much’ to ‘not bothered’)	Sociodemographic factors, health behaviors, menopausal status and symptoms	When compared to inactive women, women with >3 h of hard exercise per day were 1.5 times (1.1–1.9) more likely to report daily hot flashes		x		
de Azevedo Guimaraes et al. 2011 [45]	120 Brazilian women aged 45–59 years old volunteered for the 12-week study (recruited through work or other institutions)	Habitual PA was assessed through the short form of the International PA Questionnaire (IPAQ); Participants were classified as: maintained <30 min/day, maintained or increased to 30–60 min/day, or maintained or increased to >60 min/day	Hot flashes were assessed using the Kupperman Menopausal Index	Socio-demographic factors, anthropometrics, menopausal status and symptoms, and QOL	Women classified in the highest active group (maintained or increased to 60 min per day) had reported significantly fewer hot flashes after 12-weeks than the other two active groups after adjustment for baseline values			x	
	104 women completed the 12-week study.								
Non-randomized intervention studies									
Karacan, 2010 [50]^a^	112 women aged 46–55. The analytic sample included 65 participants that regularly participated in the 3- and 6-month exercise program	The 6-month exercise program included aerobic activity (75–80 % heart rate capacity) with calisthenics for 3 days a week for 55 min each session	The menopause rating scale (MRS) was composed of 11 items assessing menopausal symptoms divided into three groups: psychological, somatic-vegetative and urogenital	Physical characteristics (height, weight, and age at menopause), resting heart rate and blood pressure, lower back flexibility, hand grip strength, and body composition (skin folds)	There was a significant decrease in hot flushes and night sweats from baseline to 6-months			x	
Randomized controlled trials									
Agil et al. 2010 [51]	42 Turkish postmenopausal women (aged 45–60 years old) who agreed to participate in the 8-week study after presenting to the Department of Obstetrics and Gynecology (Bayindir Hospital) between March and December 2009. Participants were randomly assigned to the aerobic or resistance training group	Aerobic and Resistance Groups: Supervised sessions 3 × per week. The resistance group used elastic belt; no other details provided for either group	Vasomotor symptoms were assessed using the Menopause-specific Quality of Life Questionnaire (MENQOL)	Socio-demographics and health behaviors	Both the aerobic and resistance groups had a significant reduction in vasomotor symptoms following the exercise program.			x	
Luoto et al. 2012 [52]^a^	176 Finnish white women were recruited for the study by newspaper advertisements. The analytic sample included 154 inactive participants were randomly assigned to the exercise (*n* = 74) or control group (*n* = 77) that completed the 6-month study protocol	Exercise Group: Unsupervised aerobic training intervention; 4 × per week at 64–80 % maximal heart rate for 50 min each time	Hot flashes were assessed via the Women’s Health Questionnaire (primary). Hot flashes were also collected 2 × per day using a mobile phone-administered questionnaire	Socio-demographic factors, anthropometrics, and menopausal symptoms	WHQ assessed hot flashes did not differ by group	x			
					There was no group x time differences in daily reported daytime hot flashes.				
Moilanen et al. 2012 [53]^a^	176 Finnish white women were recruited for the study by newspaper advertisements. The analytic sample included 154 inactive participants were randomly assigned to the exercise (*n* = 74) or control group (*n* = 77) that completed the 6-month study protocol	Exercise Group: Unsupervised aerobic training intervention; 4 × per week at 64–80 % maximal heart rate for 50 min each time	The frequency of night sweats were collected 2 × per day using a mobile phone- administered questionnaire	Socio-demographic factors, anthropometrics, and menopausal symptoms	The prevalence of night sweats decreased pre- to post- intervention			x	
Newton et al. 2014 [54]^a^	Women aged 40–62 recruited from 3 sites in US (IN, CA, WA) and randomly assigned to a 12-week yoga (*n* = 107), exercise (*n* = 106), or usual activity (*n* = 142) group. Participants were and also randomly assigned to the omega-3 (*n* = 177) or placebo (*n* = 178) group. Participants were followed for 12-weeks	Yoga Group: Supervised: 1 × per week for 90 min; Unsupervised: 6 × per week for 20 min	Frequency and intensity of vasomotor were recorded in daily diaries by the participants. VMS bother was rated each day on a scale ranging from 1 ‘none’ to 4 ‘a lot’. Baseline frequency was calculated from the mean number of vasomotor symptoms reported in a 24-h period during the 14 days prior to the 1^st^ visit. Vasomotor frequency during weeks 6 and 12 were computed similarly using the corresponding diaries	Socio-demographics, anthropometrics, daily diaries assessing vasomotor symptoms, sleep quality, health history, and anxiety	After 12-weeks, based on intent-to-treat analysis, yoga had no effect on vasomotor frequency or bother when compared to usual activity	x			
		Usual Activity: Instructed to follow usual physical activity plan; asked not to initiate yoga or a new exercise regimen.							
Reed et al. 2014 [55]^a^	Women aged 40–62 recruited from 3 sites in US (IN, CA, WA) and randomly assigned to a 12-week yoga (*n* = 107), exercise (*n* = 106), or usual activity (*n* = 142) group. Participants were and also randomly assigned to the omega-3 (*n* = 177) or placebo (*n* = 178) group. Participants were followed for 12-weeks	Yoga Group: Supervised: 1 × per week for 90 min; Unsupervised: 6 × per week for 20 min	Menopausal Quality of Life Questionnaire (MENQOL; range, 1–8) is a 29-item assessment of menopause-related QOL. Total score and 4 domain-specific scores (vasomotor, physical, psychosocial, & sexual functioning). Frequency of vasomotor symptoms were also assessed via daily diaries	Socio-demographics, anthropometrics, daily diaries assessing vasomotor symptoms, sleep quality, health history, and anxiety	After 12-weeks, compared to the usual activity group, yoga group participants had significant improvements in vasomotor symptoms (as reported via MENQOL). There was no difference in pre- to post- vasomotor symptoms between the exercise and usual activity groups				x
		Exercise Group: Supervised: 3 × per week, 50–60 % HRR during month 1, 60–70 % HRR during months 2 & 3							
		Usual Activity: Instructed to follow usual physical activity plan; asked not to initiate yoga or a new exercise regimen							
Sternfeld et al. 2014 [56]^a^	Women aged 40–62 recruited from 3 sites in US (IN, CA, WA) and randomly assigned to a 12-week yoga (*n* = 107), exercise (*n* = 106), or usual activity (*n* = 142) group. Participants were and also randomly assigned to the omega-3 (*n* = 177) or placebo (*n* = 178) group. Participants were followed for 12-weeks	Exercise Group: Supervised: 3 × per week, 50–60 % HRR during month 1, 60–70 % HRR during months 2 & 3. Possible modes included, treadmill, elliptical trainer, or stationary bicycle. Trained staff recorded heart rate, workload, and perceived 7 exertion every 5–10 minutes	Frequency and intensity of vasomotor were recorded in daily diaries by the participants. VMS bother was rated each day on a scale ranging from 1 ‘none’ to 4 ‘a lot’. Baseline frequency was calculated from the mean number of vasomotor symptoms reported in a 24-h period during the 14 days prior to the 1^st^ visit. Vasomotor frequency during weeks 6 and 12 were computed similarly using the corresponding diaries	Socio-demographics, anthropometrics, daily diaries assessing vasomotor symptoms, sleep quality, health history, and anxiety	After 12-weeks, compared to the usual activity group, exercise group participants had no change in frequency or burden of vasomotor symptom, compared to the usual activity group	x			

^a^Physical activity dose reflective of 2008 Physical Activity Guidelines for Americans [3]

**Table 2:** Selected studies of physical activity and vaginal dryness

Reference	Sample	Physical activity measure	Menopausal symptom measure	Other measures	Detailed findings	Summarized findings: observed association			
						Null	Positive	Negative	Mixed
Cross-sectional studies									
Aydin et al. 2014 [34]	1071 Islamicpostmenopausalwomen (of 1328 women that expressed interest) who attended an outpatient clinic from 2005–12	Questionnaire included an item on regular exercise, defined as 30-min for ≥2 times per week (yes/no)	Validated questionnaire assessing genitourinary symptoms, including presence or absence of vaginal dryness	Socio-demographics, health behaviors, anthropometrics, length of menopausal status (months)	The prevalence of vaginal dryness was higher in participants reporting regular exercise		x		
Tan et al. 2014 [33]	305 Turkish (District of Izmir) menopausal women who went to their primary care physician between August and October 2009	International Physical Activity Questionnaire (IPAQ)-short version. Women were classified as: low, moderate, or high active	Turkish version of the Menopause Rating Scale (MRS), which includes 11 items assessing assess somato-vegetative, psychological and urogenital symptoms; scores range from ‘not present’ to ‘very severe’	Socio-demographic factors, health behaviors, anthropometrics	High active women had a lower prevalence of vaginal dryness symptoms than low and moderate active women			x	
Prospective cohort studies									
de Azevedo Guimaraes et al. 2011 [45]	120 Brazilianwomen aged45–59 years oldvolunteered for the 12-week study(recruited throughwork or other institutions)	Habitual PA was assessed through the short form of the International PA Questionnaire (IPAQ); Participants were classified as: maintained <30 min/day, maintained or increased to 30–60 min/day, or maintained or increased to >60 min/day	Vaginal dryness was assessed using the Kupperman Menopausal Index	Socio-demographic factors, anthropometrics, menopausal status and symptoms, and QOL	There was no difference in reported vaginal dryness by activity group	x			
	104 women completed the 12-week study								
Non-randomized intervention studies									
Karacan, 2010 [50]^a^	112 women aged 46–55. The analytic sample included 65 participants that regularly participated in the 3- and 6-month exercise program	The 6-month exercise program included aerobic activity (75–80 % heart rate capacity) with calisthenics for 3 days a week for 55 min each session	The menopause rating scale (MRS) was composed of 11 items assessing menopausal symptoms divided into three groups: psychological, somatic-vegetative and urogenital	Physical characteristics (height, weight, and age at menopause), resting heart rate and blood pressure, lower back flexibility, hand grip strength, and body composition (skin folds)	There was no pre- to post- exercise program difference in vaginal dryness	x			
Randomized controlled trials									
Moilanen et al. 2012 [53]^a^	176 Finnish white women were recruited for the study by newspaper advertisements. The analytic sample included 154 inactive participants were randomly assigned to the exercise (*n* = 74) or control group (*n* = 77) that completed the 6-month study protocol	Exercise Group: Unsupervised aerobic training intervention; 4 × per week at 64–80 % maximal heart rate for 50 min each time	The presence of vaginal dryness were collected 2 × per day using a mobile phone- administered questionnaire	Socio-demographic factors, anthropometrics, and menopausal symptoms	The prevalence of vaginal dryness decreased pre- to post- intervention			x	

^a^Physical activity dose reflective of 2008 Physical Activity Guidelines for Americans [3]

**Table 3:** Selected studies of physical activity and urinary incontinence

Reference	Sample	Physical activity measure	Menopausal symptom measure	Other measures	Main findings	Summarized findings: observed association			
						Null	Positive	Negative	Mixed
Cross-sectional studies									
Aydin et al. 2014 [34]	1071 Islamic postmenopausal women (of 1328 women that expressed interest) who attended an outpatient clinic from 2005–12	Questionnaire included an item on regular exercise, defined as 30-min for ≥2 times per week (yes/no)	Validated questionnaire assessing genitourinary symptoms, including presence or absence of urinary symptoms (dysuria, frequency, urgency, nocturia, and incontinence)	Sociodemographic factors, health behaviors, anthropometrics, length of menopausal status (months)	There was no significant difference in urinary symptoms in regular exercisers versus non-exercisers	x			
Prospective cohort studies									
de Azevedo Guimaraes et al. 2011 [45]	120 Brazilian women aged 45–59 years old volunteered for the 12-week study (recruited through work or other institutions)	Habitual PA was assessed through the short form of the International PA Questionnaire (IPAQ); Participants were classified as: maintained <30 min/day, maintained or increased to 30–60 min/day, or maintained or increased to >60 min/day	Urinary complaints (exertion-induced urinary incontinence or difficult micturition) assessed using the Kupperman Menopausal Index	Sociodemographic factors, anthropometrics, menopausal status and symptoms, and QOL	Women classified in the highest active group (maintained or increased to 60 min per day) had reported significantly less instances of leaking urine			x	
	104 women completed the 12-week study								
Non-randomized intervention studies									
Karacan, 2010 [50]^a^	112 women aged 46–55. The analytic sample included 65 participants that regularly participated in the 3- and 6-month exercise program	The 6-month exercise program included aerobic activity (75–80 % heart rate capacity) with calisthenics for 3 days a week for 55 min each session	The menopause rating scale (MRS) was composed of 11 items assessing menopausal symptoms divided into three groups: psychological, somatic-vegetative and urogenital	Physical characteristics (height, weight, and age at menopause), resting heart rate and blood pressure, lower back flexibility, hand grip strength, and body composition (skin folds)	There was a significant reduction in urinary symptoms from baseline to 6-months			x	
Randomized controlled studies									
Moilanen et al. 2012 [53]^a^	176 Finnish white women were recruited for the study by newspaper advertisements. The analytic sample included 154 inactive participants were randomly assigned to the exercise (*n* = 74) or control group (*n* = 77) that completed the 6-month study protocol	Exercise Group: Unsupervised aerobic training intervention; 4 × per week at 64-80 % maximal heart rate for 50 min each time	The frequency of urinary symptoms were collected 2 × per day using a mobile phone- administered questionnaire	Socio-demographic factors, anthropometrics, and menopausal symptoms	There was no change in urinary symptoms as a result of the exercise intervention	x			

^a^Physical activity dose reflective of 2008 Physical Activity Guidelines for Americans [3]

**Table 4:** Selected studies of physical activity and sleep quality and/or sleep disturbances

Reference	Sample	Physical activity measure	Menopausal symptom measure	Other measures	Detailed findings	Summarized findings: observed association			
						Null	Positive	Negative	Mixed
Cross-sectional studies									
Canário et al. 2012 [27]	Population-based sample of 370 women from Natal, Brazil aged 40–65	International Physical Activity Questionnaire with three categories of classification: sedentary, moderately active and very active (vigorous)	Blatt–Kupperman Menopausal Index with three categories of classification: mild (≤19), moderate (20–35), or severe (>35)	Socio-demographic and behavioral characteristics	Bivariate analysis revealed a statistically significant inverse association between physical activity and insomnia			x (insomnia)	
Casas et al. 2012 [38]^a^	48 month follow-up data from the Women on the Move through Activity and Nutrition (WOMAN) Study. The analytic sample included 393 postmenopausal women, aged 62 ± 3 years	Modifiable Activity Questionnaire (past year version). Participants were also classified as high or low active based on sample-determined median (11.8 MET⋅hr⋅wk^−1^)	Pittsburgh Sleep Quality Index (PSQI)	Socio-demographic factors, anthropometrics, hormone therapy status, cardiovascular risk factors	Bivariate analysis suggest that sleep quality and duration did not vary in participants classified as high vs. low active	x (sleep quality & duration)			
Lambiase et al. 2013 [39]	Sub-sample of 52 Study of Women’s Health Across the Nation (SWAN) participants (Pittsburgh site only) attending the 10^th^ annual visit (2008–09)	Kaiser Physical Activity Survey, including four indices of physical activity: (a) household/ caregiving, (b) occupational, (c) active living, and (d) sport/exercise activity. Each index was calculated as the average score (ranged from 1 to 5)	Minimitter Actiwatch-64 (dominant wrist) and sleep diary. In the diary, participants reported times in and out of bed and number of awakenings	Demographic factors, medical history, medication use, and health behaviors	Participants with higher physical activity levels reported better sleep quality and recorded fewer nighttime awakenings				x
			Participants were also asked to report their global sleep quality in the past month on a 4-point scale (very bad to very good)		Reported physical activity was not significantly associated with objectively-determined sleep estimates				
Kline et al. 2013 [40]	339 participants from the Study of Women’s Health Across the Nation (SWAN) Sleep Study, an ancillary study located at 4 of 7 SWAN clinical sites (Chicago, IL; Detroit Area, MI; Oakland, CA; Pittsburgh, PA). Data were collected from 2003–05	Kaiser Physical Activity Survey, including four indices of physical activity: (a) household/ caregiving, (b) occupational, (c) active living, and (d) sport/exercise activity. Each index was calculated as the average score (ranged from 1 to 5). Recent (KPAS scores from preceding SWAN visit) and historical (2–4 KPAS assessments in the 5–6 years prior to the SWAN Sleep Study) physical activity estimates were created. Participants were further classified as, “consistently active”, “inconsistent/ moderate” or “consistently inactive” based on the historical estimates	In-home polysomnography (PSG), daily sleep diaries, and the Pittsburgh Sleep Quality Index (PSQI)	Sociodemographic factors, medication use, menopausal status, vasomotor symptoms and other health behaviors	Higher sports/exercise index scores were significantly related with greater sleep quality and continuity (via diary) and greater sleep depth (PSG). Those with a higher sports/exercise index had a significantly lower odds of meeting diagnostic criteria for insomnia. The associations with the household or active living index were not statistically significant		x (sleep quality)		
Mansikkamäki et al. 2015 [30]	Random sample of 5000 women born in 1963 was obtained from the Finnish Population Register Centre. Analytic sample included 2606 women aged 49 years old that responded to a postal survey in 2012	A single item pertaining to usual exercise (frequency and duration) per week during past 12-months. Women were classified as ‘active’ if they reported ≥ 150 min per week of moderate intensity or ≥75 min of vigorous intensity, with strength training and balance training	Women’s Health Questionnaire addressing nine domains of physical and emotional experiences, including sleep problems	Socio-demographic factors, anthropometrics, self-rated health	There was no difference in reported sleep problems in active vs. inactive	x (sleep problems)			
Non-randomized intervention studies									
Karacan, 2010 [50]^a^	112 women aged 46–55. The analytic sample included 65 participants that regularly participated in the 3- and 6-month exercise program	The 6-month exercise program included aerobic activity (75–80 % heart rate capacity) with calisthenics for 3 days a week for 55 min each session	The menopause rating scale (MRS) was composed of 11 items assessing menopausal symptoms divided into three groups: psychological, somatic-vegetative and urogenital	Physical characteristics (height, weight, and age at menopause), resting heart rate and blood pressure, lower back flexibility, hand grip strength, and body composition (skin folds)	There was a significant decrease in reported sleeping problems from baseline to 3- and 6-months			x (sleep problems)	
Randomized controlled studies									
Kline et al. 2012 [58]^a^	437 sedentary, overweight/obese participants from the Dose–response to Exercise in postmenopausal Women (DREW) Study, randomized to no exercise (*n* = 102), 50 % (*n* = 155), 100 % (*n* = 104), or 150 % (*n* = 103) of the NIH Consensus Panel physical activity recommendations	Exercise Training Groups: The supervised exercise program (3–4 times per week) included aerobic activity at varying doses (i.e., 4-, 8-, or 12- kcal per kilogram of body weight per week (KKW). For the 1^st^ week all exercise training groups expended 4 KKW. Then, the 8- and 12- KKW groups increased energy expenditure by 1 KKW until they reached the appointed dose	Medical Outcomes Study (MOS) Sleep Scale was used to assess sleep quality during the previous 4-weeks. A modified Sleep Problems Index (SPI) was also used to assess overall sleep quality. SPI scores >25 were used to indicate significant sleep disturbance	Socio-demographic factors, anthropometric measures, medication use, health behaviors, diet, cardiorespiratory fitness, heart rate variability	After adjustment: (1) a significant effect of the intervention was found with reported sleep quality, (2) a linear dose–response effect was found with reported sleep quality across treatment groups, (3) compared to the control group, the exercise groups all had a lower odds of having significant sleep disturbance, and (4) the odds of having significant sleep disturbance decreased across increasing exercise doses		x (sleep quality)	x (sleep disturbances)	
Mansikkamäki et al. 2012 [59]^a^	176 inactive women, aged 40–63 years with no current or recent (<3 months) hormone therapy use, and 6 to 36 months since last menstruation	Exercise Program: aerobic training, 4 times per week for 50 min each time for 6-months. Participants were asked to include at least 2 sessions of walking or Nordic walking per week	Reported sleep was obtained via 1-item included on a mobile phone administered questionnaire. Participants responded to the question, “how well did you sleep last night” via 5 response options ranging from poor to good	Socio-demographic factors, health behaviors, anthropometrics	Sleep quality improved significantly more in the exercise vs. control group. The odds for sleep improvement were 2 % in the exercise group compared to −0.5 % in the control group. Women randomized to the intervention also reported significantly fewer hot flushes disturbing their sleep than the control group		x (sleep quality)		
Sternfeld et al. 2014 [56]^a^	Women aged 40–62 recruited from 3 sites in US (IN, CA, WA) and randomly assigned to a 12-week yoga (*n* = 107), exercise (*n* = 106), or usual activity (*n* = 142) group. Participants were and also randomly assigned to the omega-3 (*n* = 177) or placebo (*n* = 178) group. Participants were followed for 12-weeks	Exercise Group: Supervised: 3 × per week, 50–60 % HRR during month 1, 60–70 % HRR during months 2 & 3. Possible modes included, treadmill, elliptical trainer, or stationary bicycle. Trained staff recorded heart rate, workload, and perceived exertion every 5–10 min	Sleep quality and sleep disturbances were ascertained via the Pittsburgh Sleep Quality Index (PSQI) and insomnia symptoms were collected using the Insomnia Severity Index (ISI)	Socio-demographics, anthropometrics, daily diaries assessing vasomotor symptoms, health history, and anxiety	After 12-weeks, compared to the usual activity group, exercise group participants reported greater improvement in sleep quality and insomnia symptoms		x (sleep quality)	x (insomnia symptoms)	

^a^Physical activity dose reflective of 2008 Physical Activity Guidelines for Americans [3]

**Table 5:** Selected studies of physical activity and psychological symptoms

Reference	Sample	Physical activity measure	Menopausal symptom measure	Other measures	Detailed findings	Summarized findings: observed association			
						Null	Positive	Negative	Mixed
Cross-sectional studies									
Canário et al. 2012 [27]	Population-based sample of 370 women from Natal, Brazil aged 40–65	International Physical Activity Questionnaire with three categories of classification: sedentary, moderately active and very active (vigorous)	Blatt–Kupperman Menopausal Index with three categories of classification: mild (≤19), moderate (20–35), or severe (>35)	Socio-demographic and behavioral characteristics	Bivariate analysis revealed a statistically significant inverse association between physical activity and depression			x (depression)	
Mansikkamäki et al. 2015 [30]	Random sample of 5000 women born in 1963 was obtained from the Finnish Population Register Centre, 2606 women aged 49 years old responded that responded to a postal survey in 2012	A single item pertaining to usual exercise (frequency and duration) per week during past 12-months. Women were classified as ‘active’ if they reported ≥ 150 min per week of moderate intensity or ≥75 min of vigorous intensity, with strength training and balance training	Women’s Health Questionnaire addressing nine domains of physical and emotional experiences, including anxiety/depressed mood	Sociodemographic factors, anthropometrics, self-rated health	In the unadjusted and adjusted models, inactive women had a statistically significant increased probability of anxiety/ depression [Unadjusted POR: 1.44 (95 % CI: 1.26, 1.65); Adjusted POR: 1.31 (95 % CI: 1.14, 1.51)			x (anxiety, depression)	
Moilanen et al. 2010 [31]	Participants drawn from Finnish Health 2000 Study (*n* = 7,977), data collection included a home interview, 3 self-administered questionnaires, and a clinical exam. Analytic sample included 1427 women, ages 45–64; known menopausal status) who completed the home interview, first questionnaire	Physical activity was assessed via a single item on the questionnaire, “How much do you exercise or strain yourself physically in your leisure time” with four response options ranging from ‘sedentary’ (reading, watching television) to ‘competitive sports’. Participants were classified based on low, moderate, and high physical activity	Severity of general symptoms, including psychological symptoms (e.g., depression), were assessed via two items on the questionnaire	Socio-demographics, health behaviors, anthropometrics, menopausal status and hormone therapy use	Compared to the high active group, low active women were significantly more likely to report psychological symptoms			x (psychological symptoms)	
Timur et al. 2010 [35]	Community-based randomly selected sample of 685 Turkish (Malatya) women aged 45–59 years. Data were collected from February to May, 2008	A single item to assess regular exercise, operationalized as:≥3 times per week or not (yes or no)	The Beck Depression Inventory, a 21 question survey that uses a Likert scale from 0 to 3 to assess severity of depressive symptoms	Socio-demographics, anthropometrics, health behaviors, parity, menopausal status and hormone therapy use	No significant difference in depression by regular exercise status	x			
Vallance et al. 2010 [36]^a^	297 post-menopausal women from the Palliser Region of Alberta, Canada	Godin Leisure-Time Exercise Questionnaire which assesses the frequency and duration of mild-, moderate-, and strenuous- leisure-time physical activity	Depression was assess via the 20-item Center for Epidemiologic Studies-Depression scale. For each item, responses ranged from 0 ‘<1 day in the past week’ to 3 ‘5-7 days in the past week’	Socio-demographic factors, anthropometrics, health history, menopausal symptoms	Unadjusted and adjusted analyses found that participants meeting physical activity recommendations reported significantly fewer depression symptoms than those who did not			x (depression symptoms)	
		Participants also wore a pedometer (DigiWalker SC-01) for 3 days, average steps per day were computed	Anxiety was assessed via the 10-item Spielberger’s state Anxiety Inventory (SAI). For each item, responses ranged from 1 ‘not all’ to 4 ‘very much so’						
		Estimates reflecting meeting physical activity recommendations were also computed for both reported and pedometer-based (>7500 steps per day) estimates.							
Chang et al. 2013 [37]	Secondary data analysis of 481 multi-racial/ethnic women who completed questions on menopausal symptoms that were part of a larger Internet survey study	Kaiser Physical Activity Survey, including four indices of physical activity: (a) household/ caregiving, (b) occupational, (c) active living, and (d) sport/exercise activity. Each index was calculated as the average score (ranged from 1 to 5)	Midlife women’s Symptoms Index, which measured psychological symptoms based on their prevalence ‘yes’ or ‘no’ and severity ‘1 = not at all and 5 = extremely’	Sociodemographic factors, self-rated health, menopausal status, hormone therapy use	After adjustment, there was a statistically significant association between the household/ caregiving index and psychological symptoms in Non-Hispanic Asians and Blacks, only. Associations were not statistically significant for any other race/ethnic group or indices of physical activity				x
Prospective cohort studies									
Dugan et al. 2015 [46]	Included 2891 participants from the Study of Women’s Health Across the Nation. Women were recruited in 1995–97. Included data from follow-up, 3, 6 & 9	Kaiser Physical Activity Survey, including four indices of physical activity: (a) household/ caregiving, (b) occupational, (c) active living, and (d) sport/exercise activity. Each index was calculated as the average score (ranged from 1 to 5). Participants were then classified as: meeting physical activity guidelines, below physical activity guidelines or Inactive	Depression was assess via the 20-item Center for Epidemiologic Studies-Depression scale. For each item, responses ranged from 0 ‘<1 day in the past week’ to 3 ‘5–7 days in the past week’. High depressive symptoms were classified as ≥16	Socio-demographic factors, health behaviors, anthropometrics, menopausal status, hormone therapy use, antidepressant medication use	After adjustment for covariates, participants classified as ‘meeting physical activity guidelines’ or ‘below guidelines’ had a significantly lower odds for depressive symptoms than those classified as inactive. This association persisted over 10 years of observation			x (depressive symptoms)	
de Azevedo Guimaraes et al. 2011 [45]	120 Brazilian women aged 45–59 years old volunteered for the 12-week study (recruited through work or other institutions)	Habitual PA was assessed through the short form of the International PA Questionnaire (IPAQ); Participants were classified as: maintained <30 min/day, maintained or increased to 30–60 min/day, or maintained or increased to >60 min/day	Psychological symptoms were assessed using the World Health Organization Quality of Life Brief Version Questionnaire; higher scores reflect less severe psychological symptoms	Socio-demographic factors, anthropometrics, menopausal status and symptoms, and QOL	Women classified in the highest active group (maintained or increased to 60 min per day) had increased psychological domain QOL scores after 12-weeks than the other two active groups after adjustment for baseline values		x (better psycho-social symptoms)		
	104 women completed the 12-week study								
Non-randomized Intervention Studies									
Karacan, 2010 [50]^a^	112 women aged 46–55. The analytic sample included 65 participants that regularly participated in the 3- and 6-month exercise program	The 6-month exercise program included aerobic activity (75–80 % heart rate capacity) with calisthenics for 3 days a week for 55 min each session	The menopause rating scale (MRS) was composed of 11 items assessing menopausal symptoms divided into three groups: psychological, somatic-vegetative and urogenital	Physical characteristics (height, weight, and age at menopause), resting heart rate and blood pressure, lower back flexibility, hand grip strength, and body composition (skin folds)	There was a significant reduction in psychological symptoms, including depressive mood, irritability, and anxiety after 3- and 6-months of the exercise program. Reported exhaustion also significantly decreased from baseline to 3- and baseline to 6- months			x (psychosocial symptoms)	
Randomized Controlled Studies									
Agil et al. 2010 [51]	42 Turkish, postmenopausal women aged 45–60 years old, presented to the Department of Obstetrics and Gynecology of Bayindir Hospital between March and December 2009 and volunteered to participate in an 8-week physical activity intervention. The analytic sample included 36 participants; intent to treat analysis was not done	Participants were randomly assigned to either an aerobic (*n* = 18) or resistance (via elastic bands) (*n* = 18) physical activity intervention. Both groups were supervised, 3 days per week. No other details were provided	Menopause Rating Scale (MRS) assessed psychological symptoms, the Beck Depressive Inventory (BDI) was used to assess depressive symptoms	Socio-demographic factors, health behaviors	Psychological symptoms decreased significantly in both groups post exercise programs according to the MRS subscale. The BDI showed a decrease in depressive symptoms for both groups, but was higher in the resistance exercise group than the aerobic exercise group			x (psychosocial symptoms)	
Moilanen et al. 2012 [53]^a^	176 Finnish white women were recruited for the study by newspaper advertisements. The analytic sample included 154 inactive participants were randomly assigned to the exercise (*n* = 74) or control group (*n* = 77) that completed the 6-month study protocol	Exercise Group: Unsupervised aerobic training intervention; 4 × per week at 64–80 % maximal heart rate for 50 min each time	The frequency of psychological symptoms (i.e., mood swings, depressive moods, irritability) were collected 2 × per day using a mobile phone- administered questionnaire	Socio-demographic factors, anthropometrics, and menopausal symptoms	The prevalence of mood-swings decreased pre- to post- intervention. No other reductions were noted				x
Sternfeld et al. 2014 [56]^a^	248 women aged 40–62 recruited from 3 sites in US (IN, CA, WA) and randomly assigned to a 12-week yoga (*n* = 107), exercise (*n* = 106), or usual activity (*n* = 142) group. Participants were and also randomly assigned to the omega-3 (*n* = 177) or placebo (*n* = 178) group. Participants were followed for 12-weeks	Exercise Group: Supervised: 3 × per week, 50–60 % HRR during month 1, 60–70 % HRR during months 2 & 3. Possible modes included, treadmill, elliptical trainer, or stationary bicycle. Trained staff recorded heart rate, workload, and perceived exertion every 5–10 min	Depressive symptoms were assessed using the Patient Health Questionnaire-8 (PHQ-8) and anxiety symptoms using the Generalized Anxiety Disorder-7 (GAD-7)	Socio-demographics, anthropometrics, daily diaries assessing vasomotor symptoms, sleep quality, and health history	Compared to the usual activity group, the exercise group had a greater decrease in depressive symptoms (*p* = 0.028), but did not meet the set alpha level of *p* < 0.0125 for multiple comparisons. Change in anxiety symptoms did not differ between the exercise and usual activity groups				x
Villaverde Gutiérrez et al. 2012 [57]^a^	330 postmenopausal women, aged 60–70, were recruited from a healthcare clinic in Granada, Spain. Of those, 60 (19.1 %) meet eligibility criteria and were willing to participate. Women were randomly selected to the exercise (*n* = 30) or control (*n* = 30) group and followed for 6-months. Three women from the exercise group were excluded for not completing at least 80 % of the exercise intervention	Exercise group: During the first 8 weeks of the supervised program, 2 × per week, 50 min each time, 50–70 % heart rate reserve. During weeks 8–12, 3 × per week, 60 min each time, 50–70 % heart rate reserve and muscle training exercises were added. Weeks 12–24, intensity was increased to 60–85 % heart rate reserve; all other components were similar to weeks 8–12	Depressive symptoms were assessed via the 30-item Geriatric Depression Scale (GDS). Participants were classified as: moderate depression (11–14) or severe depression (15–30). Anxiety was assessed via the 14-itemHamilton Anxiety Scale (HRSA). Responses ranged from 0 ‘absence of symptoms’ to 4 ‘total incapacitated’. Participants were classified as: minor anxiety (6–15) or major anxiety (>15)	Anthropometrics	Unadjusted results suggest that among the exercise group, women initially classified with moderate or severe depression had significantly reduced depressive symptoms after 6-months. Similarly, participants in the exercise group, classified with minor or major anxiety had significantly reduced anxiety symptoms after 6-months. In the Control group, women initially classified with moderate depression had a slight increase in depressive symptoms after 6 months. This slight increase was also shown in the control group among participants initially classified with minor anxiety			x (severe depression, depressive symptoms & anxiety)	
		Control group: Received no exercise treatment							

^a^Physical activity dose reflective of 2008 Physical Activity Guidelines for Americans [3]

**Table 6:** Selected studies of physical activity and weight gain

Reference	Sample	Physical activity measure	Menopausal symptom measure	Other measures	Detailed findings	Summarized findings: observed association			
						Null	Positive	Negative	Mixed
Prospective cohort studies									
Choi et al. 2012 [47]	346 women, aged 40–50 years with regular menstrual cycles were enrolled in the Biobehavioral Health in Diverse Midlife Women Study in 1996–1997. The analytic sample included 232 pre (*n* = 175) and peri (*n* = 57) menopausal women that completed physical activity data at baseline and after 2 years	Paffenbarger Physical Activity Questionnaire was assessed every 6-months for 2 years. Leisure time physical activity estimates are MET · hr · wk^−1^ and are computed as the product of the duration and frequency, weighted by the corresponding MET value for each reported activity. After 2-years, change physical activity status was classified as: increase (≥300 MET · hr · wk^−1^), maintain (−300 to 300 MET · hr · wk^−1^), or decrease (<300 MET · hr · wk^−1^)	Trained study staff measured body weight (via electronic scale) and waist circumference (specialized tape to the nearest 0.1 cm), every 6 months	Sociodemographic factors and Menopausal status (via urinary levels of FSH)	Unadjusted results suggest that after 2-years, participants who maintained their physical activity had an average weight gain of 3.3 ± 12.2 lbs. Participants who decreased physical activity gained the most weight over time 5.3 ± 8.9 lbs. Participants who increased physical activity gained the least amount of weight 0.8 ± 12.2 lbs. Similar group differences were also shown for waist circumference. Compared to those who decreased physical activity over time, those that increased physical activity had statistically significant less weight gain (*p* < 0.05) and waist circumference increase (*p* < 0.01), after adjustment for covariates			x	
Lusk et al. 2010 [48]	18,414 Nurses’ Health Study (NHS) II participants, recruited in 1989. Follow-up questionnaires including physical activity and body weight were completed every 2-years. The analytic sample participants who were premenopausal through 2005 and completed the 1989 and 2005 questionnaires	The NHS II Physical Activity Questionnaire includes reported frequency and duration (10 response options from ‘zero’ to ‘≥11 h per week’ of 9 specific activity types over the past year. Usual walking pace was also reported (responses range from ‘unable to walk’ to ‘very brisk (≥4 miles per hour). Average number of flights of stairs climbed daily were also reported. Inactivity via reported sitting time was also assessed	Height and weight were participant reported On the baseline and follow-up questionnaires. BMI was computed from these self-reported values	Socio-demographic factors, dietary patterns (i.e., sugar-sweetened beverages, trans-fats, and dietary fiber), health behaviors, parity, oral contraceptive use, antidepressant use	A 30 min per day increase in overall physical activity levels between 1989 and 2005 was associated with less weight gain [−1.31 kg (95 % CI: −1.44, −1.18)]. A 30 min increase in brisk walking and bicycling, specifically, was associated with less weight gain [−1.81 kg (95 % CI: −2.05, −1.56) and −1.59 kg (95 % CI: −2.09, −1.08), respectively]. Further, women that reported no bicycling in 1989 and increased to ≥5 min per day in 2005, gained significantly less weight [−0.74 (95 % CI: −1.41, −0.07)] than those who reported no bicycling in 2005			x	
Sims et al. 2012 [49]	Participants were drawn from the Women’s Health Initiative (WHI) Study (40 clinical sites) and included 58,610 postmenopausal women aged 50–79 years old that took part in either the diet modification or hormone therapy arms. Participants enrolled in 1993–98 and were followed annually for 8 years	The WHI Physical Activity Questionnaire includes reported frequency and duration within moderate- and strenuous- physical activity categories. Walking was also assessed. Participants were further classified into four groups: sedentary (≤100 MET · hr · wk^−1^), low activity (>100 to 500 MET · hr · wk^−1^), moderate activity (>500 to 1200 MET · hr · wk^−1^), and high activity (≥1200 MET · hr · wk^−1^)	Trained clinical staff measured body weight and height with a calibrated balance beam or digital scale and a wall-mounted stadiometer. BMI was calculated from these measures. Waist (midpoint between last floating rib and upper part of the iliac crest at the end of expiration)-to-hip (maximum extension of the buttocks) ratio (WHR) was also measured using a conventional measuring tape	Sociodemographic factors, dietary intake, smoking, alcohol, hormone use, and sleep	In the fully adjusted models, in the 50–59 year age group, women in the moderate activity group experienced a significant weight loss [−0.30 (95 % CI: −0.53, −0.07) compared to the sedentary group. In women aged 70–79 years, higher physical activity was significantly associated with less weight loss [0.34 (95 % CI: 0.04, 0.63)			x	
Non-randomized intervention studies									
Karacan, 2010 [50]^a^	112 women aged 46–55. The analytic sample included 65 participants that regularly participated in the 3- and 6-month exercise program	The 6-month exercise program included aerobic activity (75–80 % heart rate capacity) with calisthenics for 3 days a week for 55 min each session	Height and weight were assessed with a metal meter and scale; BMI was also computed. Body fat percentage was also measured via skinfold calipers using the Sloan and Weir formula (triceps and suprailiac)	Menopausal symptoms, physical characteristics (age at menopause), resting heart rate and blood pressure, lower back flexibility, hand grip strength, and body composition (skin folds)	There was a significant decrease in body weight, BMI, and body fat percentage from baseline to 6-months			x	

^a^Physical activity dose reflective of 2008 Physical Activity Guidelines for Americans [3]

### Potential biological mechanisms: physical activity and menopausal symptoms

Physical activity has both acute and chronic physiological and psychological effects, many of which could help to alleviate menopausal symptoms and other complaints of midlife women. Even though the specific etiology of vasomotor symptoms remains unclear, hot flashes and night sweats are the result of neuroendocrine processes at the level of the hypothalamus [60]. One hypothesis for how physical activity might alleviate vasomotor symptoms is through the impact of physical activity on neurotransmitters (e.g., β-endorphins) which regulate thermoregulation [61]. Similarly, physical activity, which increases sympathetic nervous system activity, could alleviate the vaginal dryness which results from the declines in circulating estrogen characteristic of menopause [62] by increasing sexual arousal and lubrication [63]. However, it is unclear if this is an acute effect of physical activity or if the increased lubrication persists at rest. The benefit of physical activity for reduced risk of urinary incontinence is likely mediated through obesity. Previous studies have indicated that obesity is a risk factor for urinary incontinence and studies have shown that weight loss can result in urinary incontinence remission [64]. The mechanisms by which physical activity may improve sleep quality include associated reductions in anxiety and depression. More directly, physical activity has been shown to promote increases in slow wave sleep, which is indicative of good sleep quality. Physical activity may also impact sleep through favorable influences on circadian functioning [65]. As reported by Dugan et al., the proposed biological mechanisms supporting the beneficial role of physical activity for preventing or reducing depression include: reduced inflammation, increased neurotransmitter (i.e., dopamine and serotonin) levels, and increased endorphin secretion [46]. Finally, physical activity contributes to prevention of weight gain and promotion of weight loss and reduces risk of adiposity-related outcomes because physical activity is a key component of total energy expenditure (i.e., ~20 % of total energy expenditure) [66].

### Physical activity and vasomotor symptoms

Table 1 summarizes the recent evidence examining the association between physical activity and vasomotor symptoms, including hot flashes and night sweats. In a 15-day longitudinal study, Elavsky et al. [41] found an acute bout of exercise (30 min of moderate intensity exercise) decreased subjective and objectively determined hot flashes, but had no impact on night sweats. Also, daily physical activity estimates (detected via accelerometry during the 15-day observation period) were not associated with reported hot flash frequency, although less fit participants reported more hot flashes on days when they engaged in more activity than usual. In another longitudinal study by Elavsky and colleagues [42], participants concurrently wore an accelerometer and reported daily hot flashes via an electronic personal digital assistant for 30 consecutive days. Statistically significant same-day and cross-lagged (previous day’s physical activity compared to hot flashes the next day) associations were highly variable in both magnitude and direction. Three recent prospective cohort studies have also been conducted, including one showing a null association [43], another showing an increased risk of hot flashes among women classified as active [44], and the third reporting significantly fewer hot flashes in women classified in the highest active group (i.e., maintained or increased to >60 min per day over 12-weeks) [45]. A non-randomized intervention study also reported a significant decrease in reported hot flashes and night sweats following a 6-month aerobic program [50], and the same general finding was seen in a small randomized control trial of Turkish women (*n* = 42) [51].

In contrast, the evidence from the majority of randomized controlled trials, including results from the 2 × 3 Factorial Menopause Strategies: Finding Lasting Answers for Symptoms & Health (MsFLASH) Study, shows no association between physical activity and vasomotor symptoms [54, 56]. For MsFLASH, women were recruited from three sites: Indianapolis, IN, Oakland, CA, and Seattle, WA and were randomized (3:3:4) to 12 weeks of exercise, yoga, or usual activity and further randomized to (1:1) to omega-3 fish oil or a placebo. Women in the yoga group performed one, 90 min session of supervised yoga per week and 20 min of unsupervised yoga on all other days. The exercise group participated in an individualized, supervised aerobic program (3 times per week, 40–60 min per session) with a progressively increasing energy expenditure goal. During month 1, the prescribed workload was 50–60 % of heart rate reserve. In months 2 and 3, heart rate reserve was increased to 60–70 % heart rate reserve [55]. Activity modes included: treadmill, elliptical trainer, or stationary bike. Heart rate and perceived exertion was recorded every 5–10 min by trained supervisors [56]. The usual activity group were instructed to follow their usual activity patterns and were asked to not begin a yoga or new exercise program [55, 56]. Reed et al. [55] reported that after the 12-week program, the yoga group had significant improvements in reported vasomotor symptoms, obtained via the 29-item Menopausal Quality of Life Questionnaire (MENQOL), when compared to the usual activity group. However, when the frequency and intensity of vasomotor systems were obtained using more sophisticated daily diaries, yoga had no effect on the vasomotor symptoms when compared to the usual activity group [54]. This null association was also found when comparing the reported frequency and burden of vasomotor symptoms via daily diaries between the exercise and usual activity groups [56]. This evidence from the more rigorous RCT studies largely supports the 2014 Cochrane Report by Daley et al. [67], which concluded there was insufficient evidence to demonstrate that physical activity is an effective treatment for management of vasomotor symptoms.

### Physical activity and vaginal dryness

As shown in Table 2, the recent evidence from cross-sectional studies examining the association between physical activity and vaginal dryness is mixed [33, 34], while a prospective cohort study [45] of Brazilian women by de Azevedo Guimaraes and colleagues found no association between habitual physical activity and vaginal dryness. Similarly, a non-randomized intervention study found no pre- to post- exercise program difference in vaginal dryness after 6-months [50]. Yet, in a randomized controlled trial of Finnish women [53] the prevalence of vaginal dryness decreased pre- to post intervention following a 6-month, unsupervised aerobic training program (4 times per week, 50 min per session at 64–80 % of maximal heart rate) in the treatment group versus control.

### Urinary incontinence

Table 3 presents the evidence regarding the association between physical activity and urinary symptoms, including incontinence. In the prospective cohort study of Brazilian women by de Azevedo Guimaraes et al., women classified in the highest active group (maintained or increased to >60 min per day), reported less instances of leaking urine than those classified as low or moderately active at the 12-week follow-up [45], and in the non-randomized intervention study by Karacan [50], there was a reduction in urinary symptoms following a 6-month aerobic exercise program. However, in the Finnish randomized controlled study, there was no change in urinary symptoms as a result of a 6-month aerobic exercise program [53]. When interpreting these findings it is important to note that associations were not adjusted for change in body weight, which is unfortunate given the proposed underlying biological mechanism between physical activity and urinary incontinence.

### Sleep quality and disturbances

Cross-sectional studies have generally shown better sleep quality and/or fewer sleep disturbances among physically active women (Table 4); this association was also shown in the non-randomized intervention study by Karacan [50] and has been largely confirmed in recent evidence from randomized controlled trials. Utilizing data from the Dose–response to Exercise in postmenopausal Women (DREW) Study [58], participants randomized to any of the three exercise groups reported improvements in sleep quality when compared to the control group. Further, a dose–response effect was shown with reported sleep quality across the exercise groups, with the magnitude of the effect increasing with each increase in exercise dose. The exercise intervention arms were designed specifically to reflect 50 %, 100 % or 150 % of the National Institutes of Health (NIH) Consensus Panel physical activity recommendations [68]. Further, the odds of reporting a significant sleep disturbance were also lower with a dose response relation in all exercise groups compared to the controls. The beneficial effect of physical activity on sleep quality was also shown in two additional randomized control studies, including the Finnish study [59] and the MsFLASH trial [56]. The Advisory Committee for the development of the *2008 Physical Activity Guidelines for Americans* [2], concluded that the evidence supporting the benefit of physical activity for improved sleep quality was moderate. These findings will likely provide additional support for the next iteration of the *Guidelines*.

### Psychological distress: depression and anxiety

As shown in Table 5, the majority of the recent evidence in midlife women supports an inverse association between physical activity and depressive symptoms, including anxiety. Indeed of six recent cross-sectional studies [27, 30, 31, 35–37], only one study [35] found no difference in depressive symptoms by regular exercise status (≥3 times per week). In the prospective cohort study of Brazilian women, de Azevedo Guimaraes et al. [45], found improved psychological symptoms after 12-weeks in the high active group. This was also shown in an analysis of SWAN participants. Here, those classified as meeting physical activity guidelines had a lower odds of depression than inactive participants and this finding persisted over 10 years [46]. Karacan [50] also reported a reduction in psychological symptoms, including depressive mood, irritability, and anxiety after 3 and 6 months of participation in an aerobic exercise program. There was also a statistically significant reduction in exhaustion from baseline to 3 and 6 months. Of the four studies detailing findings from randomized controlled studies, all demonstrated a beneficial effect of physical activity for psychological symptoms including depressive symptoms [51, 56, 57], mood swings [53], and anxiety symptoms [57] when compared to a control [51, 53, 57] or usual activity group [56]. However, the impact of the physical activity intervention on depressive symptoms did not reach statistical significance in the MsFLASH Study [56] due to a more conservative alpha level to account for multiple comparisons (α = *p* < 0.028). These findings generally support the conclusions of the *2008 Physical Activity Guidelines for Americans* [2] Advisory Committee that rated the evidence pertaining to the benefit of physical activity for reduced risk of depression as strong.

### Weight gain

Table 6 outlines the recent evidence including three prospective cohort studies [47–49] and one non-randomized intervention study [50], supporting an inverse association between physical activity and weight gain Cross-sectional studies were not included in this review because the outcome was weight change or weight loss over time,. In a study by Choi and colleagues [47], 346 participants from the Biobehavioral Health in Diverse Midlife Women Study, the 2-year change in physical activity was categorized as increase, decrease, or maintained. Participants who increased physical activity levels had significantly less weight gain and less of an increase in waist circumference when compared to those who decreased physical activity levels, after controlling for age, initial physical activity and relevant outcome value (both *p* < 0.05). Similarly, in an analysis of Nurses’ Health Study II participants [48], a 30-min increase in leisure-time physical activity levels between 1989 and 2005 was significantly associated with less weight gain [−1.31 kg (95 % CI: −1.44, −1.18)], and these same findings were found for the associations with weight change and walking and bicycling, specifically [−1.81 kg (95 % CI: −2.05, −1.56) and −1.59 kg (95 % CI: −2.09, −1.08), respectively]. In an analysis of 58,610 Women’s Health Initiative participants [49], the associations between physical activity groups (sedentary, low-, moderate-, and high- active) and weight change were examined by age group (50–59 years, 60–69 years, and 70–79 years). Interestingly, Sims et al., reported that among the youngest age group, women in the moderate activity group experienced a significant weight loss [−0.30 (95 % CI: −0.53, −0.07) compared to the sedentary group. Yet, in women aged 70–79 years, higher physical activity was associated with the attenuation of the expected age-related weight loss due to loss of lean mass observed in this age group [0.34 (95 % CI: 0.04, 0.63). Authors posit that this attenuation in weight loss was due to the retention of lean muscle mass rather than a loss in adipose tissue [49]. Finally, Karacan [50] reported a significant decrease in body weight, body mass index (BMI), and body fat percentage (via skinfolds) after a 6-month supervised, aerobic-based physical activity program. However, these associations were not adjusted for potential confounders or other covariates. These findings generally support the conclusions of the *2008 Physical Activity Guidelines for Americans* [2] Advisory Committee that rated the evidence supporting the benefit of physical activity for the prevention of weight gain and promotion of weight loss as strong, particularly when combined with reduced dietary intake. There is also currently moderate to strong evidence to support the inverse association between physical activity and abdominal adiposity.

## Conclusions

The recent evidence, accumulated over the past 5 years, regarding the association between physical activity and hormone-related (i.e., primary) menopausal symptoms in midlife women generally mirrors previous research in this area in that the evidence remains either null or inconclusive. However, with more general health outcomes that result from biological aging, including poor sleep quality, increased depressive symptoms, and weight gain, the evidence supporting the beneficial effect of physical activity is quite conclusive. For primary menopausal symptoms, the inconsistencies across studies may be due to differences in targeted study populations. (i.e., study eligibility based on general age range, reflecting midlife versus menopausal status) as well as measurement strategies used to assess physical activity and menopausal symptom outcomes. Further, many studies did not examine and/or report the observed physical activity-menopausal symptom associations by menopausal status. This is particularly important given that the prevalence and severity of reported symptomology varies by menopausal status, as reported by Woods et al. [17]. Finally, for studies including a physical activity intervention component, there have also been distinct differences in the specific targets in terms of prescribed activity mode (i.e., aerobic versus resistance), frequency, intensity, and duration.

While it is intuitive that physical activity measurement strategies may vary across studies due to differences in the target population (e.g., race and cultural differences, menopausal status), a preponderance of studies included in this review utilized physical activity questionnaires with unreported and/or unknown measurement properties. This is despite recently published evaluation studies demonstrating the test-retest reliability and validity of physical activity questionnaires designed specifically for midlife women [69]. It is well-established that physical activity behaviors in women are quite different than in men and can vary by activity domain and/or preferred activity type [70]. For example, midlife women may accumulate the majority of their daily physical activity in domestic pursuits (e.g., caretaking) and walking or yoga during leisure-time. Therefore, it is critically important that, whenever possible, physical activity questionnaires used in this population are structured to elicit the most accurate information (i.e., reliable and valid) on the types of physical activities that are most pertinent to midlife women. This practice was implemented in a few studies included in this review that utilized more established questionnaires including, the International Physical Activity Questionnaire (IPAQ), Kaiser Physical Activity Survey (KPAS), or Modifiable Activity Questionnaire (MAQ) was used. A few additional studies included in this review used accelerometers, with known measurement properties, to quantify the physical activity exposure [41, 42].

Another weakness is that several observational studies included in this review, classified participants into physical activity groups in analyses, and did not provide details on the threshold limits used to distinguish groups. While these categories, distinguishing non-exerciser from exerciser or low and moderate active from high active, may have acceptable internal study validity, the categorization is a substantial limitation to interpreting overall study findings within the context of the entire body of literature relevant to physical activity and menopausal symptoms during midlife. Further, the practice of utilizing cut-point thresholds that are not meaningful from a clinical or public health standpoint may increase the likelihood for potential misclassification bias of the physical activity exposure and also lead to spurious findings.

Since there is currently a lack of evidence regarding the specific dose of physical activity that confers menopausal symptom risk reduction, threshold values used for analysis should be based on meaningful categories that reflect current physical activity recommendations for general health benefit [3]. This same practice should also be applied when designing physical activity interventions. The specific physical activity targets or components of interventions should allow participants to accumulate at least 150 min of moderate intensity physical activity per week to reflect current physical activity guidelines [3]. However, it is important to note that midlife women may also have pre-existing disease or disability that may preclude their ability to fully meet recommended physical activity levels. For these women, even low to moderate increases in daily physical activity may be beneficial to health, which is also noted in the *2008 Physical Activity Guidelines for Americans* [3]. Further, the intervention should include activity modes or types that are common and acceptable among midlife women, including brisk walking or bicycling. These intervention specific details should be included in the methods section of all peer-reviewed publications to facilitate the interpretation of the study findings. The MsFLASH [54–56] and DREW studies [58] provide excellent examples of how best to implement these recommendations when designing and/or reporting findings from physical activity intervention studies.

In summary, the recent evidence has not provided much clarity regarding the role of physical activity with menopausal symptoms in mid-life women beyond what was already known [67]. Yet, the evidence supporting the beneficial role of physical activity for more general health outcomes, including sleep quality, psychological distress, and weight gain, is quite conclusive. Given the considerable prevalence of sleep disturbances [71], depressive symptoms [72, 73], and overweight/obesity [74] in midlife women, health care and physical fitness professionals should encourage their patients or clients to engage in regular physical activity levels to reduce risk of these important health outcomes. For some midlife women, this may be sufficient “*reward*” to overcome the “*risk*” of allocating sufficient time, in an already busy schedule, to be physically active. In addition, midlife is a particularly vulnerable period when individuals are at immediate risk for disability, and there is moderate to strong evidence to support the beneficial role of physical activity for optimizing functional health and reducing risk of falls among older adults [3]. However, midlife women initiating a new exercise program should strive to make small, incremental increases in physical activity levels over time to reduce risk of acute musculoskeletal injuries, including sprains and strains [3]. Finally, while the evidence is still accumulating regarding the role of physical activity for specific menopausal symptoms, health care professionals should periodically remind midlife women that they will experience a reduced lifetime risk of chronic disease and disability development if they remain physically active as they age.
